# Deformative stress associated with an abnormal clivo-axial angle: A finite element analysis

**DOI:** 10.4103/2152-7806.66461

**Published:** 2010-07-16

**Authors:** Fraser C. Henderson, William A. Wilson, Stephen Mott, Alexander Mark, Kristi Schmidt, Joel K. Berry, Alexander Vaccaro, Edward Benzel

**Affiliations:** Doctors Community Hospital, Georgetown University Hospital, United States; 1 Computational Biodynamics LLC., Yale University, United States; 2 Dartmouth-Hitchcock Medical Center, Georgetown University Hospital, United States; 3 Bethesda MRI & CT, Georgetown University Hospital, United States; 4 University of Alabama Medical Center, Georgetown University Hospital, United States; 5 Kettering University, United States; 6 Thomas Jefferson University Hospital, United States; 7 Cleveland Clinic Foundation, United States

**Keywords:** Chiari malformation, clivo-axial angle, craniocervical junction, deformative stress, finite element analysis, stretch myelopathy

## Abstract

**Background:**

Chiari malformation, functional cranial settling and subtle forms of basilar invagination result in biomechanical neuraxial stress, manifested by bulbar symptoms, myelopathy and headache or neck pain. Finite element analysis is a means of predicting stress due to load, deformity and strain. The authors postulate linkage between finite element analysis (FEA)-predicted biomechanical neuraxial stress and metrics of neurological function.

**Methods:**

A prospective, Internal Review Board (IRB)-approved study examined a cohort of 5 children with Chiari I malformation or basilar invagination. Standardized outcome metrics were used. Patients underwent suboccipital decompression where indicated, open reduction of the abnormal clivo-axial angle or basilar invagination to correct ventral brainstem deformity, and stabilization/ fusion. FEA predictions of neuraxial preoperative and postoperative stress were correlated with clinical metrics.

**Results:**

Mean follow-up was 32 months (range, 7-64). There were no operative complications. Paired *t* tests/ Wilcoxon signed-rank tests comparing preoperative and postoperative status were statistically significant for pain, bulbar symptoms, quality of life, function but not sensorimotor status. Clinical improvement paralleled reduction in predicted biomechanical neuraxial stress within the corticospinal tract, dorsal columns and nucleus solitarius.

**Conclusion:**

The results are concurrent with others, that normalization of the clivo-axial angle, fusion-stabilization is associated with clinical improvement. FEA computations are consistent with the notion that reduction of deformative stress results in clinical improvement. This pilot study supports further investigation in the relationship between biomechanical stress and central nervous system (CNS) function.

## INTRODUCTION

Ventral brainstem compression (VBSC) from basilar invagination is a well-known cause of encephalomyelopathy in the setting of Chiari malformation[1–6] and in platybasia.[7–9] Most of these patients have traditionally undergone suboccipital decompression and expansile duraplasty, but up to a half either failed to show improvement or exhibited a delayed regression despite decompression.[2,5,10,11] In the latter instance, posterior decompression often exacerbates cervicomedullary angulation over the odontoid process.[3] The importance of this angulation of the brainstem, or “medullary kinking,” has been oft cited.[4,6,11–17] Sawin *et al*. refer to the “fulcrum effect in basilar invagination, by which traction is applied to the caudal brainstem and rostral cervical spinal cord, producing prominent bulbar dysfunction and myelopathy.”[6] Kubota demonstrated that a clivo-axial angle of less than 130° was associated with delay or failure to recover after foramen magnum decompression.[18] Numerous series of patients report cervicomedullary kyphosis or ventral flattening in the presence of a kyphotic clivo-axial angle or retroflexed odontoid process.[2,5,6,16] Medullary kinking and basilar invagination introduce abnormal deformative stresses in the brainstem and spinal cord,[19–23] which result in neurobiological changes that are believed to underlie the pathophysiology of many of the observed neurological changes in this group of patients.[12–15,22–32]

In a novel approach, the authors applied a finite element analysis (FEA) research tool to compute estimates of preoperative and postoperative mechanical stress within the brainstem and spinal cord in 5 children with medullary kinking due to kyphotic clivo-axial angulation in the context of Chiari malformation or basilar invagination. These stresses were compared with clinical metrics.

Finite element analysis is a mathematical method that reduces a continuous structure into discrete finite brick elements. This method allows the approximation of partial differential equations with a linear system of ordinary differential equations, which can then be solved by numerical methods with the appropriate boundary conditions. In this particular case, the equations concern mechanical strain, out-of-plane loading and material properties such as Young’s modulus of elasticity or Poisson’s ratio.

A model of the brainstem and spinal cord that incorporates patient-specific anatomical data such as deformity over the odontoid process, lengthening of brainstem and spinal cord with flexion, and numerous other features such as compression of the spinal cord by a herniated disc or spur has been developed to parametrically generate specific finite element models for each patient. The computations derived from these models undergoing flexion and extension generate estimates of the stresses existing within the brainstem and spinal cord in the neutral, flexion and extension conditions. The estimated stresses reflect the dynamic change in stress exerted on the neural tissue. The importance of biomechanical stress has recently been demonstrated in the neurobiological, clinical, experimental and biomechanical literature.

The FEA estimations of deformative strain, generated postoperatively, were used to test the hypothesis that reduction of abnormal stresses improved neurological deficits. The 5 patients studied herein underwent open reduction (normalization of the clivo-axial angle) and posterior translation to normalize the craniospinal relationship. This reduction was followed by occipitocervical fusion and stabilization.[1,7,16] Correlation of computed mechanical stresses with clinical outcome indices suggested a direct relationship between reduction of deformative stress and clinical improvement.

## MATERIALS AND METHODS

From 2003 to 2007, 5 children with encephalomyelopathy due to medullary kinking, basilar invagination or Chiari malformation were evaluated by a pediatric neurologist and referred for neurosurgical evaluation. The study was IRB approved for neurological assessment, evaluation of quality of life (SF-36), American Spinal Injury Association (ASIA) impairment scale, pain (Visual Analog Scale [VAS], Oswestry Neck Disability Index), function (Karnofsky Index) and assessment of bulbar symptoms (the Brainstem Disability Index — 20 questions relating to bulbar symptoms, Table 1), and computational brainstem and spinal cord stress injury analysis (SCOSIA©, Computational Biodynamics, LLC, Va Beach, VA). All data, with the exception of the ASIA scale, were collected independently by the research assistant while the surgeon was not present. Surgical rationale determination and clinical decision–making options were not included in the IRB approval process.

**Table 1:** Brainstem Disability Index

The following 20 symptoms may be referable to pathology at the level of the brainstem. Please indicate yes or no whether your child has any of the following symptoms on a recurring or chronic basis.
Double vision
Memory loss
Dizziness
Vertigo
Ringing in the ears
Speech difficulties
Difficulty swallowing
Sleep apnea
Snoring or frequent awakening
Choking on food
Hands turn blue in cold weather
Numbness in your arms and shoulders
Numbness in your back and legs
Gets tired very easily
Unsteady walking
More clumsy than you used to be Urinates more often (every 1-2 hours)
Irritable bowel disease or gastroesophageal reflux disease
Weaker than you would expect in your arms or hand
Weaker in your legs
5% for each positive response, 0%-100%



### Rationale for surgery

The following surgical criteria were used in the deliberation as to whether subjects were candidates for surgery: first, signs of cervical myelopathy (sensorimotor findings, hyper-reflexia); second, bulbar symptoms (lower cranial nerve dysfunction, respiratory disorder, changes in vision or tracking, auditory vestibular symptoms, dysautonomia) listed in Table 1; third, severe headache and/ or neck pain that was improved by the use of a neck brace; and fourth, the radiographic finding of brainstem deformation due to Chiari malformation, basilar invagination and/ or ventral brainstem compression, as determined by the Grabb-Oakes criterion[2] and by the presence of abnormal clivo-axial angulation (<135°) from the criteria of Van Gilder and Menezes,[4,17] of Goel[1,33] of Kim, Rekate, Klopfenstein and Sonntag,[16] and of Kubota.[18] Grabb *et al*. found that the Basion - posterior C2 measurement was useful to determine which patients with Chiari malformation should undergo reduction of ventral brainstem deformity, fusion and stabilization. We used the B-pC2 measurement as an indication of ventral deformative stress regardless of the presence of Chiari malformation.

Each of the 5 patients studied were placed in a neck brace for at least 2 weeks prior to surgery to determine whether immobilization improved their clinical presentation; all showed significant improvement of clinical symptoms while in the brace. The response to the neck brace represented a subjective indicator that immobilization in a neutral or slightly extended position lessened the headache and/ or neck pain.

### Surgical procedure

Each patient was positioned prone in a Mayfield head-holder with extension at the cervicothoracic junction and gentle flexion at the craniocervical junction to facilitate both subperiosteal exposure of the subocciput and upper two or three vertebrae and placement of the suboccipital plate. Sensory and motor evoked potentials were monitored throughout. A suboccipital craniectomy was performed to the extent necessary to decompress the Chiari malformations, but with care to leave available bone surface area for the subsequent fusion. A suboccipital plate (Altius™, Biomet, Parsippany, NJ) was contoured to the occiput and fastened to the skull with screw lengths appropriate to the bone thickness as determined by preoperative CT scan. At the midline (the “keel”), the skull thickness was approximately 10 mm. Laterally, the mantle is thinner, usually accommodating a 6-mm screw.

The surgeon considered but did not perform occipito-ganglial neurectomies[1] because there were no cases of severe basilar invagination.

Open intraoperative reduction of the craniocervical junction deformity was performed *under fluoroscopy, evoked potential monitoring and direct visual inspection*, and in the manner described by Kim, Rekate and Sonntag[16] and by Goel.[1] Traction was utilized to the extent necessary to achieve reduction of basilar invagination. To accomplish this, the surgeon broke from scrub, and taking hold of the Mayfield head-holder from the head of the table, performed a three-part maneuver: first, the cranium was placed in approximately 15 lb of traction; second, a posterior translational force was applied to bring the Basion more in line with the odontoid; third, the cranium was extended at the craniocervical junction to reduce the clivo-axial angle, thereby restoring the clivus to a normal relationship with the odontoid process and eliminating the medullary kyphosis.[34] Severe basilar invagination may require preoperative traction reduction[17] or more forceful traction intraoperatively.[1,33] After normalization of the clivo-vertebral relationship, the Mayfield head-holder was re-tightened. Fluoroscopy was performed to confirm an increase (normalization) in the clivo-axial angle (CAA) of 20°. Ideally, a CAA of over 160° would be achieved. In most cases, the reduction technique was repeated to maximize normalization of the clivo-axial angle. The technique of repeated reduction to gain further improvement of the clivo-axial angle takes advantage of the viscoelastic properties of the ligamentous structures that stabilize the craniovertebral junction and upper cervical spine. Craniospinal stabilization was completed utilizing screws placed in the C1 lateral mass and in the C2 pedicles. The technique for screw placement in the C1 lateral mass was described by Goel.[1,35,36] The surgeon must exercise caution because the vertebral artery foramen lies medially in 30% of cases, and may fall within the standard trajectory of the C1 lateral mass screw. In these cases, a single screw may be placed. When additional screw purchase was deemed necessary, the C3 lateral masses were added as points of fixation. Subjects with Ehlers-Danlos syndrome who manifest significant joint laxity should have C3 lateral mass screws included in the construct. The bone surfaces were decorticated; segments of two ribs were harvested,[111] contoured to the suboccipital bone and upper cervical vertebrae, and augmented with demineralized bone matrix. Both wounds were then closed over drains. The patients were mobilized in a neck brace (Miami J™ or equivalent) for 6 weeks.

### Finite element analysis

An FEA program (PRIMEGen) was adapted for the purpose of modeling the brainstem and cervical and upper thoracic spinal cord under dynamic loading and strain. The resulting Spinal Cord Stress Injury Analysis (SCOSIA^©^) technology computes probable magnitude and location of stress within the brainstem and upper spinal cord. The Von Mises stress is the aggregate of both in-line strain, or stretching; and the stress due to “out-of-plane loading,” such as from odontoid compression.

Computer-driven stress analysis–based finite element formulations provide a unique perspective on the biomechanical behavior of the human cervical spine under normal, degenerative and iatrogenically surgically altered conditions. Due to the reproducibility and repeatability of finite element models, detailed parametric analysis with regards to the geometrical conditions and material property changes can be performed, and biomechanical responses can be evaluated using FEA. FEA is routinely used to study spine mechanics.[37–47] More recently, FEA has been applied to spinal disorders[48] and spinal cord pathologies.[20,21,49]

Due to the displacement-based formulation of structural finite elements, nodal displacements are primary output variables and nodal stresses are computed variables using nodal displacements. In other words, stresses are predicted based upon the deformation or stretching of specific nodes, with specific Cartesian coordinates within the system.

**Figure 1:**
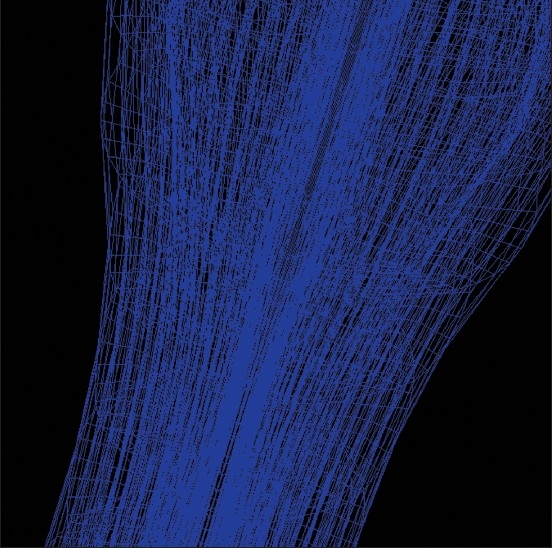
Generation of a finite element mesh

The SCOSIA system utilizes a simplified model of the brainstem and spinal cord, assuming isotropy for gray matter tracts and for the white matter tracts, constant material properties regardless of stress, boundary conditions at the pons and mid thorax, and Young’s modulus of elasticity for bovine gray and white matter provided by Ichihara *et al*.[50] Upright MRIs and surface coils would have been preferable but were not available during this study; instead, cervical spine MRI in the neutral position was used to determine “out-of-plane” loading such as arises from deformity, retroflexed odontoid, discs and spurs. Dynamic flexion/ extension x-rays were performed in the upright position to model strain due to change in length, and for the generation of the “centroids,” the x, y, z coordinates of the center of every level of the spinal cord, which go into the modeling process. Figure 1 shows the program in the process of generating the mesh.

**Figure 2:**
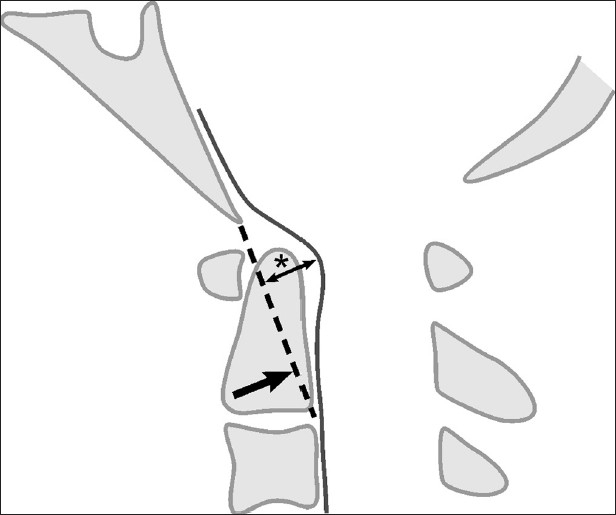
Grabb-Oakes measurement: the perpendicular distance (*) from the BpC2 line (Basion to posterior inferior C2 body) to the dura. A value greater than or equal to 9 mm indicates ventral brainstem compression

The Grabb-Oakes measurement was used to determine degree of focal compression due to VBSC.[2] On MRI, a line was drawn from the Basion to the tip of the posterior inferior C2 vertebra. A perpendicular was drawn from the B-pC2 line to the dura as shown [Figure 2]. A measurement (Δ) of greater than 9 mm reflected some degree of VBSC thus in our boundary conditions:

VBSC = Δ – 9, in mm

The acquired images were transferred to the dedicated processing workstation via DICOM; for each anatomical level, anatomical coordinates were manually specified to assemble the model of the spine. Following generation of the model, boundary conditions were imposed by fixing the model at the T6 level, displacing it into the flexed position that the patient’s spinal cord assumed as determined by flexion x-rays, and adding out-of-plane loading to the medulla equivalent to the VBSC number described above. The analysis yielded the overall Von Mises stress for each voxel within the model: σ = 3J_2_, where J_2_ is the second deviatoric stress invariant. A more detailed description of how the finite element method computes Von Mises stresses may be found in the work of Huebner *et al*.[51] For purposes of this study, only the maximal Von Mises stress (aggregate of strain and out-of-plane loading) for each component was selected. Stresses representing motor skills were taken from computed stresses within the corticospinal tract, sensation from the dorsal columns, and respiratory function from the nucleus solitarius and dorsal motor nucleus.

### Imaging

Standard parameters for diagnosing basilar invagination have been defined for MRI.[52] Two patients had a Chiari malformation; 1, basilar invagination; 1, retroflexion of the odontoid; and 1, functional cranial settling.[53] All 5 patients were observed to have ventral brainstem compression by the Grabb-Oakes criteria, an abnormal clivo-axial angle and kinking of the medulla. Spinal abnormalities included assimilation of the atlas, atlanto-axial subluxation, Klippel-Feil malformation, scoliosis and kyphosis.

### Bulbar symptoms index

Bulbar symptoms previously described[2,4,5] were indexed as another metric reflecting bulbar pathology [Table 1]. The authors included a numerical representation of bulbar symptoms (20 symptoms, 5% each for a total score of 100%; 0% reflecting no bulbar pathology; 100%, 20 bulbar symptoms). The symptom of decreased memory was included as a bulbar symptom because many of the patients have reported the onset of memory difficulties with the other symptoms, and because there is support in the literature that memory is affected with alterations of the brainstem reticular activating system,[54,55] sleep abnormalities,[56] altered visual tracking or modulation of audition,[57,58] and chronic pain.[59]

### Statistical analysis

Analyses were performed preoperatively and at the last follow-up postoperatively. Due to small sample size, both parametric (paired *t* tests) and nonparametric (Wilcoxon signed-rank tests) statistical tests were used for SF-36 physical component summary (PCS) scores and mental component summary (MCS) scores, VAS pain scores, summed ASIA scores, Karnofsky Index, Bulbar Symptoms Index, and SCOSIA-derived stress values. Pearson’s correlation coefficient (r_p_) was used to determine the extent to which SCOSIA-derived stress values were correlated preoperatively and postoperatively with VAS, brainstem disability indices, Karnofsky values and SF-36 scores. Statistical significance was set at *P* = .05.

## RESULTS

Two males and 3 females, ages 8-17 years, were followed for 24 to 64 months (mean follow-up, 36 months). The presenting diagnoses, radiological findings, overall clinical outcome and complications are listed in Table 2. Comorbidities included behavioral disorders (4/5); respiratory disorders, including sleep apnea (3/5), GERDS (2/5), scoliosis (1/5), tongue-thrusting (1/5). All 5 children had medullary kinking due to an abnormal clivo-axial angle (mean clivo-axial angle, 126°).[16,17,60] All 5 patients had 1 to 3 mm of ventral brainstem compression using the Grabb-Oakes criterion.[2] The associated symptoms and signs are presented alongside the outcome metrics, before and after surgery [Table 3]. Postoperative follow-up was 100%.

**Table 2:** Surgical series

Patient	Age (yrs)	Primary Diagnoses	CAA* pre/post	B-pC2**	Outcome	Complications	F/up (mos)
#1	9	Episodic respiratory difficulty, Chiari malformation	115°/152°	10 mm	+++	Ø	52
#2	13	Encephalomyelopathy, Basilar invagination	116°/140°	10 mm	+++	Ø	48
#3	17	Encephalomyelopathy, Basilar invagination	132°/142°	11 mm	+++	Ø	19
#4	13	Myelopathy, Chiari malformation, Scoliosis	129°/139°	11 mm	++	Ø	17
#5	15	Severe neck pain, Cranial settling	136°/161°	9 mm	+++	Ø	24

CAA* = Preoperative and postoperative clivo-axial angle;

B-pC2** = Grabb-Oakes measurement of VBSC, Basion to ventral inferior C2

**Table 3:** Preoperative and postoperative outcomes: clinical findings, metrics

Patient	Presenting symptoms and signs	Postop. symptomatic improvement	SCOSIA stress forces (N/cm^2^) preop./ postop.	Pain (×/100) preop./ postop.	Brainstem disability index preop. / postop.	ASIA preop. / postop.	Karnofsky Preop. / Postop.	SF-36 Preop. / Postop.
#1	h/o resp. arrest, asthma, sleep apnea, HA, neck pain, nausea,, anhydrosis Decreased gag reflex, dysdiadochokinesia, Babinski	- Resolution of neck pain and HA, resp. abnormalities	70/26 70/6 70/33	70/0	80%/0	M-90/M-100 P-112/P-112 Lt-112/Lt-112	50%/100%	Phys: 31/69.1 Mental: 22.9/65.2
		- Improvement in strength, coordination								
		- No change in anhydrosis							
#2	Tongue thrusting, myoclonic spasms, paresthesias weakness, tics, anisocoria, absent gag reflex, hemi-hypoesthesia, Babinski	Resolution of tongue thrusting, tics, myoclonic spasms, strength and sensory deficit	60/13 60/13 46/33	90/60	55%/5%	M-80/M-100 P-84/P-112 U-84/P-112	50%/l 00%	Phys: 43/47.6 Mental: 37.3/46.8
	Schizotypal disorder								
#3	Hyperactive, sleep apnea, HA, weakness imbalance, incoordination, urinary freq. hand flapping	- Resolution of hyperactivity, normal strength, coordination, balance, urinary urgency and hand flapping paresthesias	26/6 33/6 60/20	55/0	55%/0	M-93/M-100 P-112/P-112 Lt-112/Lt-112	85%/100%	Phys: 43.5/60.4 Mental: 47.3/57.2
	Hypoesthesia Hyper-reflexia, Babinski	- Improved hypopnea, reflexes							
	ADHD							
#4	Chronic HA, emesis, dysphagia	Resolution of scoliosis, emesis,	33/13 40/13 26/26	70/0	55%/0	M-100/M-100 P-75/P-75 Lt-75/Lt-75	80%/100%	Phys: 37.2/59.5 Mental: 30.5/58.5
	Scoliosis, paresthesia, abnormal gait	Improved gait, and dysphagia.						
	Cat-eye syndrome	Recurrence HA at 6 months. Repeat suboccip craniectomy						
#5	Emesis, dysphagia ataxia, freq. falls, vertigo, chronic neck and back pain, sleep walking, hyperactivity,	Resolution of HA, back pain, sleep walking, emesis, dysphagia and vertigo.	33/13 60/13 40/20	35/0	40%/10%	M-100/M-100 P-56/P-112 Lt-56/Lt-l 12	80%/100%	Phys: 43.7/46 Mental: 53.5/55.4
	Paresthesias, UE, fatigue, hyper-reflexia	Normalization of sensation, strength, coordination, socialization						
	Asperger’s syndrome	No fatigue						

*The preoperative and postoperative predicted stress analyses given in N/cm^2^ represent the Von Mises stress (the aggregate of stress and strain in all axes, resulting from stretch and deformation). The Von Mises stresses are shown for the corticospinal tract, the dorsal columns and the nucleus solitarius/ dorsal motor nucleus, respectively. Only the maximum stress for each tract is listed.

Preoperative and postoperative B-pC2 measurements were read independently by a neuroradiologist (A.M.) and are presented in Table 2. Postoperatively, the Grabb-Oakes measurements (Δ) were less than 9 mm, demonstrating reduction of preoperative VBSC.

### Symptoms

All patients presented with the following symptoms: headache or neck pain, weakness in the upper or lower extremities, sensory changes (hypoesthesias or paresthesias in the upper and lower extremities), clumsiness with frequent falls, uncertain gait, fatigue, gastroesophageal disturbance (reflux or irritable bowel syndrome), respiratory disturbance (including respiratory arrest [pt. #1]), and other respiratory disorders which manifested as sleep apnea, snoring or history of frequent awakening. Most reported vestibular, auditory or visual disturbance and bowel and/ or bladder dysfunction. Trophic changes, including abnormal response of circulation to cold weather or profuse sweating, occurred in only 1 patient [Table 3]. Every child reported substantial improvement in most symptoms within the first postoperative month. This improvement was sustained in every patient over the duration of follow-up, with one exception. Patient # 4, though substantially improved, suffered a recurrence of headaches at 6 months postoperatively. This is considered under “complications of surgery.” Patient # 1 reported total resolution of his respiratory events; the other children or their families reported improved sleep and resolution of snoring, frequent awakenings and nightmares.

### Signs

Preoperative neurological findings included weakness (especially hand weakness), poor muscle tone and poor posture, sensory changes, hyper-reflexia and dysdiadochokinesia. One patient was observed to have scoliosis. Sensory changes (hypoesthesia to pinprick) were never painful or unpleasant, and were frequently ignored or not recognized by the patient. The gag reflex was decreased or absent in all subjects, though usually not associated with dysphagia [Table 3].

Postoperatively, strength, sensation and posture improved in 1 month. Patient # 2 improved from mild weakness to normal strength. The scoliosis resolved to normal within the first month in patient # 4. Four of the 5 patients are performing at academic and athletic levels above their preoperative state. Substantial behavioral improvement was reported by the parents of the 4 subjects with neurobehavioral disorders, but measurement of behavior was beyond the scope of this study.

### Clinical metrics

Metrics were obtained from the subjects and their parents by a research technician (I.M.). Visual analog pain was reduced from a preoperative mean of 64 (on a “0 to 100” scale) to a postoperative mean of 12 (t= 6.15, *P*= .0002 for parametric; V= 15, *P*= .029 for nonparametric). SF-36 physical component summary (PCS) scores improved following surgery (mean, 40-57). These improvements were statistically significant (t= –2.59, *P*= .030 for parametric; *P*= .031 for nonparametric) and postoperatively were above the normal mean (sample mean = 56.5 versus normal mean = 50 ± 10 SD). Mental component summary (MCS) scores also improved (mean, 38-57), with significance (t= –2.48, *P*= .033 for parametric; *P*= .031 for nonparametric). Summed ASIA scores increased from a mean of 268.2 to a mean of 309.2, though this increase failed to achieve significance on both parametric and nonparametric tests (t= –1.83, *P*= .071 for parametric; *P*= .050 for nonparametric). The bulbar symptom index showed significantly fewer bulbar symptoms following surgery (preop. mean= 57%, postop. mean= 30%; t = 6.78, *P*= .001 for parametric; V= 15, *P*= .029 for nonparametric). Karnofsky scores significantly improved (mean, 69 preop. to 100 postop., t= –3.97, *P*= .008 for parametric; *P*= .028 for nonparametric).

### Stress modeling

SCOSIA-derived stress values paralleled the patients’ clinical conditions [Figures 3a–3d]. For instance, high stress values in the motor tracts of the brainstem and spinal cord signaled weakness. Following surgery, the 68% decrease in calculated stresses within the corticospinal tracts was congruent with the improved motor performance, as seen in the ASIA scores (*P*= 0.004/0.027). The same was true for sensory symptoms, where stress decreased by an average of 81% in the dorsal columns (*P*= 0.002/ 0.028); and for symptoms referable to the respiratory function and gastrointestinal (GI) function (irritable bowel syndrome [IBS], gastric reflux or GERDS), where stresses in the nucleus solitarius decreased by 45% (*P*= 0.021/0.05). Resolution of thoracic scoliosis in subject # 4 was concordant with decreased stress in the ventral gray matter of the upper thoracic spinal cord (from 60 N/cm^2^ to 5 N/cm^2^).

With every clinical metric, higher preoperative stress values correlated with greater disability (r= 0.36 to 0.72), lower Karnofsky values (r= –0.43 to –0.98) and lower physical component summary scores (r= –0.34 to –0.60). Correlations between stress values and mental component summary scores were more variable (r= 0.21 to –0.69). The low sample size (*n*= 5) for these cross-patient comparisons implied that most of these correlations approached, but did not achieve, statistical significance. A very strong correlation between computed stress in the corticospinal tract and Karnofsky score (which achieved significance at r= –0.98, *P*= .003) was observed.

The analysis of within-patient changes in SCOSIA estimates of neuraxial stress and patient condition metrics yielded similar results. Patients exhibiting larger decreases in SCOSIA-derived stress values experienced proportionate decreases in disability (r= 0.36 to 0.52), increases in Karnofsky values (r= –0.08 to –0.99) and increases in PCS (r= –0.22 to –0.35) and MCS scores (r= –0.10 to r= –0.37). The relationship between changes in corticospinal stress and changes in Karnofsky values was strong (r= –0.99, *P*= .001).

**Figure 3a:**
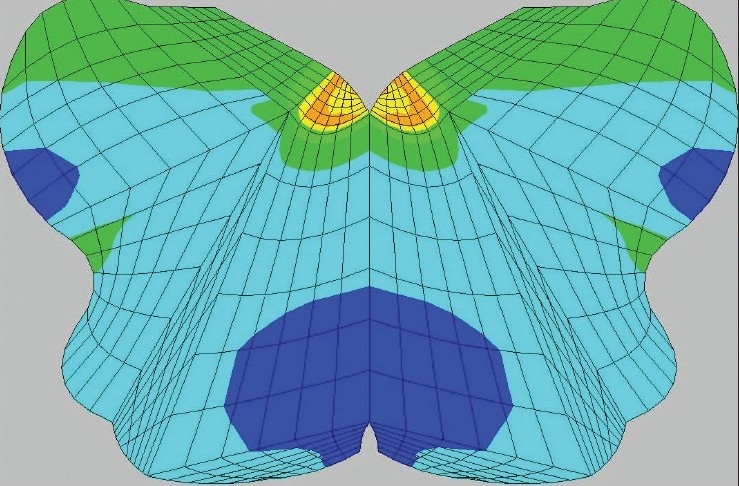
The computational predictions of stress of brainstem and spinal cord preoperatively demonstrated areas of high stress: 45-60 N/cm^2^ in the upper medulla within the region of the dorsal motor nucleus and nucleus solitarius, possibly contributive to the mild sleep apnea and the abdominal pain

**Figure 3b:**
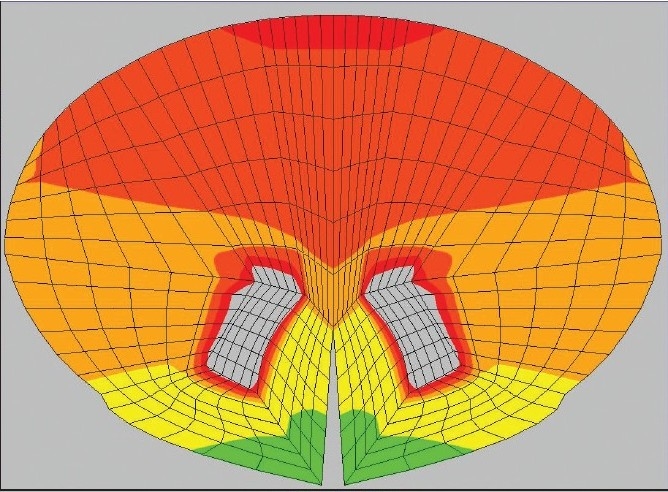
The axial view through C2 shows high stress (45-60 N/cm^2^) in the posterior and lateral columns, correlating with the widespread sensory changes, hyper-reflexia and Babinski sign. Even higher stress (70 N/cm^2^) is seen in the anterior gray matter, possibly underlying the tongue thrusting on presentation of the patient

**Figure 3c:**
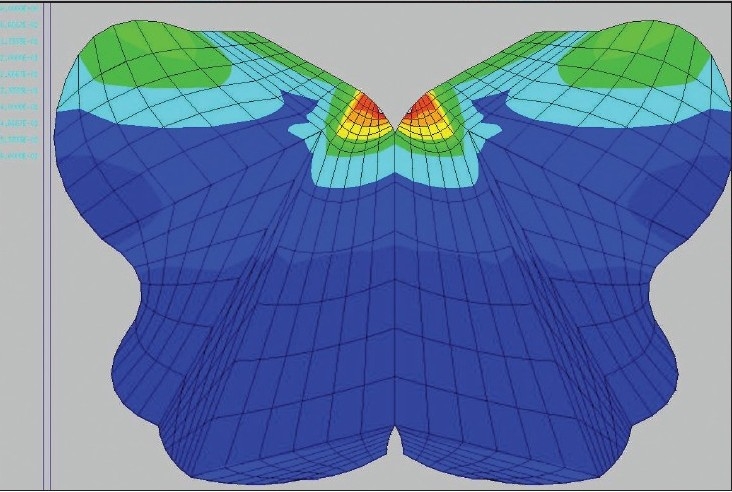
Postoperatively, the computational analysis shows reduction of stress concordant with the resolution of the patient's findings: 33 N/cm^2^ in the nucleus solitarius/ dorsal motor nucleus region of the medulla

**Figure 3d:**
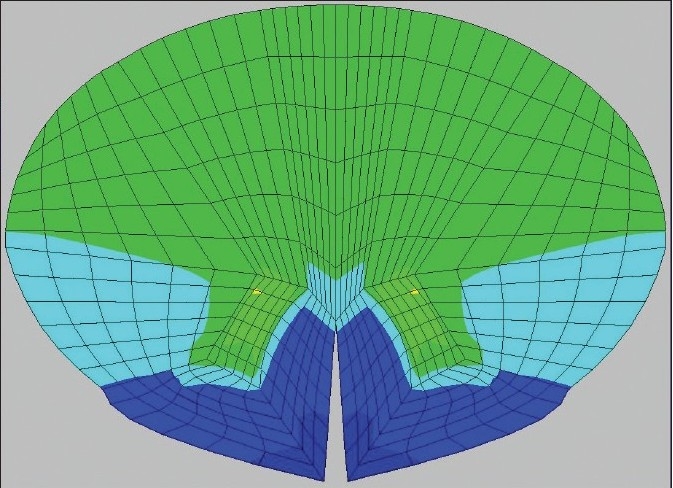
The stress has reduced at the C2 level in the posterolateral columns (13 N/cm^2^) and similarly in the anterior gray matter (13 N/cm^2^). The reduction in stress correlated with clinical improvement

### Surgical complications

There were no neurological deficits resulting from surgery, and no wound problems. Subject # 4 (with a history of craniosynostosis, and cat-eye syndrome) had undergone a limited suboccipital craniectomy for Chiari malformation. Six months later, headaches recurred and were suspected to represent occipital neuralgia. The patient’s family refused diagnostic block of the occipital nerve. The surgeon (F.C.H.) sent the child for evaluation of craniosynostosis; and, later, monitoring of ICP, which was normal (<10 cm H_2_0). Eighteen months after surgery, the parents sought enlargement of the suboccipital craniectomy; at 3 years, the headaches appear to have resolved.

### Fusion/stabilization

**Figure 4:**
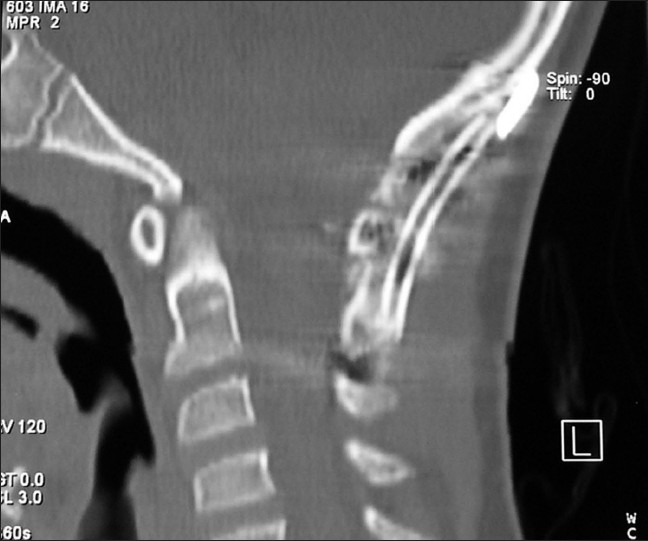
Patient # 1, status at 3 months post suboccipital craniectomy fusion/ stabilization with rib autograft. Clivo-axial angle was increased from 115° to 152°, the patient resumed non-contact sports 6 months after surgery

No subject required blood transfusion. The average duration of surgery was 3.5 hours. All subjects were discharged within 3 days after surgery. Subjects were placed in hard cervical collars (Miami J™ collars). CT scans at 3 months showed bone fusion in every case [Figure 4]. There were no hardware failures. No subjects required revision. Though cervical range of movement was decreased, only 1 subject (# 1, whose parents had been overly protective and insisted that he not move his neck for 6 months) complained of limited neck rotation. The remainder reported no complaints of limitation of range of motion at 1 year.

## DISCUSSION

### Deformative stress in the brainstem and upper cervical spinal cord

Mechanical compression at the cervicomedullary junction occurs in Chiari 1 malformation,[2,6,10,16,61,62] achondroplasia[63–65] ; or as a result of basilar invagination, clival hypoplasia, anterior indentation of the pons, upward displacement of the brainstem or anterior displacement of the foramen magnum.[3,6,16,24,66,67] Scoville and Sherman first opined that angulation of the brainstem in basilar invagination caused neurologic signs and disability.[68] Breig emphasized that craniospinal flexion increases strain in the brainstem, thus rendering it susceptible to injury.[24] Van Gilder reported that a clivo-axial angle of less than 150° was associated with neurological changes.[17] Menezes reported progression of disability in many patients following suboccipital decompression for Chiari 1, and attributed the observed bulbar findings to the fulcrum effect of the medullo-spinal junction draped over the dens.[3] Milhorat observed that retroflexion of the odontoid process in 96/364 patients and basilar invagination in 44/364 patients resulted in kinking of the medulla in 140 of 364 patients.[5] Grabb, Mapstone and Oakes noted failure of suboccipital decompression to relieve the symptoms of Chiari syndrome in 48% of the pediatric and 28% of the young adult patients, and attributed this failure to flattening of the ventral brainstem.[2] Patients with a Grabb-Oakes measurement of greater than or equal to 9 mm were considered to be at high risk for VBSC, and underwent transoral odontoidectomy. Kubota *et al*. found that patients with cervicomedullary syndromes who failed to improve after suboccipital decompression had an average clivo-axial angle of 121.7°, while those who improved rapidly had an average angulation of 142.8°.[18] Kim *et al*., Goel, Botelho and others have demonstrated that normalization of the clivo-axial angle from an average of 127° to 147° by intraoperative manipulation (extension of the clivus with respect to the odontoid to normalize the clivo-axial angle) is an effective treatment for basilar invagination, with demonstrable improvement in neurological function,[7,16,39] thus obviating the need for transoral odontoidectomy.

The patients in this series were referred for disabling neurological symptoms, which included headaches, bulbar findings and myelopathy. All subjects shared abnormality of the clivo-axial angle. The authors consider the CAA to be a surrogate measure of deformative stress in the brainstem and upper spinal cord. Neuraxial strain is accentuated with flexion of the craniocervical junction [Figures 5a–5d]. Approximately 22° of flexion/ extension occurs at the craniocervical junction.[70] Upon flexion at the craniocervical junction, there is a lengthening of the brainstem and upper spinal cord (16), which may reach pathological strains in the context of an abnormally acute CAA, or retroflexed odontoid.[71] The addition of “out-of-plane” loading greatly increases the overall Von Mises stress. As opposed to the in-line strain that occurs with stretching of the spinal cord upon flexion, “out-of-plane” loading is any deformative stress that occurs horizontally upon the neuraxis, due to indentation from deformity, stenosis or disc or horizontal strain from the dentate ligaments perpendicular to the axis of the neuraxis. Compression from the sides of a viscoelastic cylinder, such as the medulla/ upper spinal cord, will create increased longitudinal tension within the neuraxis and perpendicular to the plane of compression.[13] Thus a ventral compression force, like the retroflexed odontoid,[5] “nontraditional” basilar invagination,[16] platybasia[17,33] and “functional cranial settling” with hereditary connective tissue disease,[53] results in increased intra-axial tension. Breig’s cadaveric models showed fissuring on the side opposite the compression.[24]

**Figure 5a:**
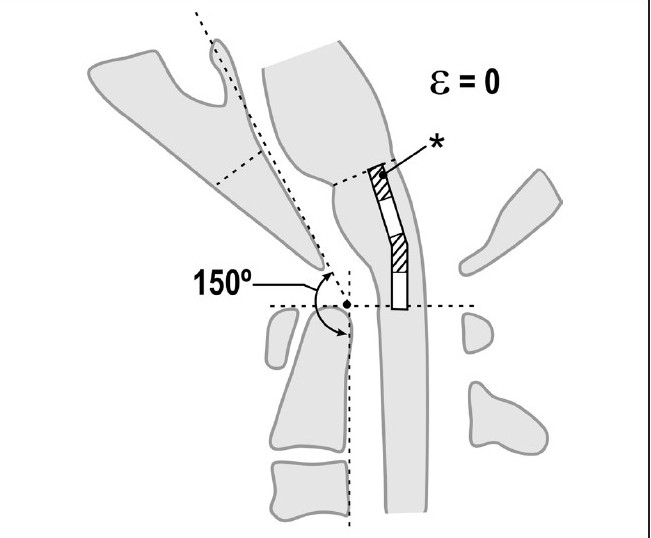
Normal craniocervical junction in the neutral position. The clivo-axial angle varies from 150° to 165°. There is minimal neuraxial strain in the neutral state

**Figure 5b:**
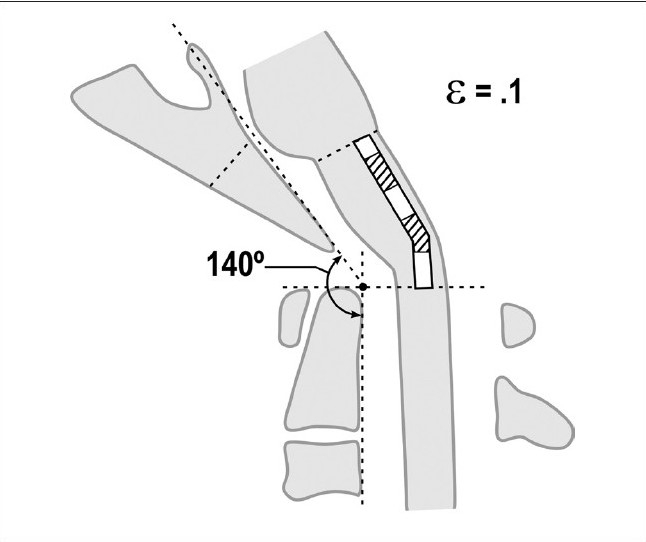
Normal craniocervical junction in flexion. The neuraxis stretches by approximately 10% of its total length with flexion of the craniocervical junction creating a strain ε = 0.1

**Figure 5c:**
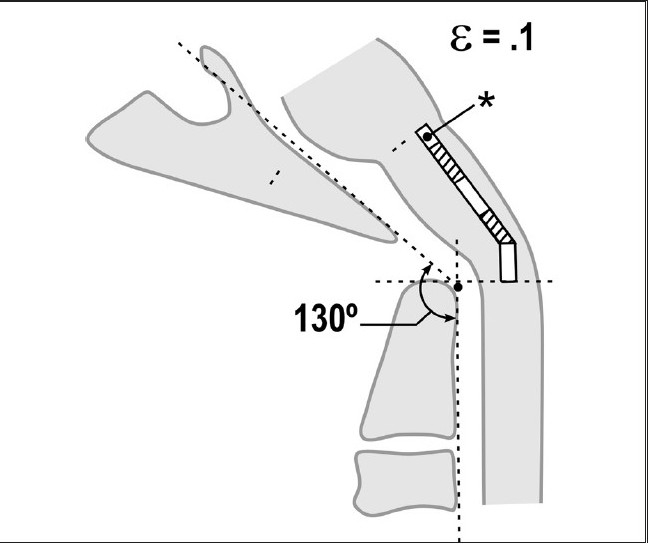
Pathological craniocervical junction with an abnormal clivo-axial angle in the neutral position. As a result of medullary kyphosis due to retroflexion of the odontoid, basilar invagination or other forms of ventral brainstem compression or deformity, the resting strain is ε = 0.1

**Figure 5d:**
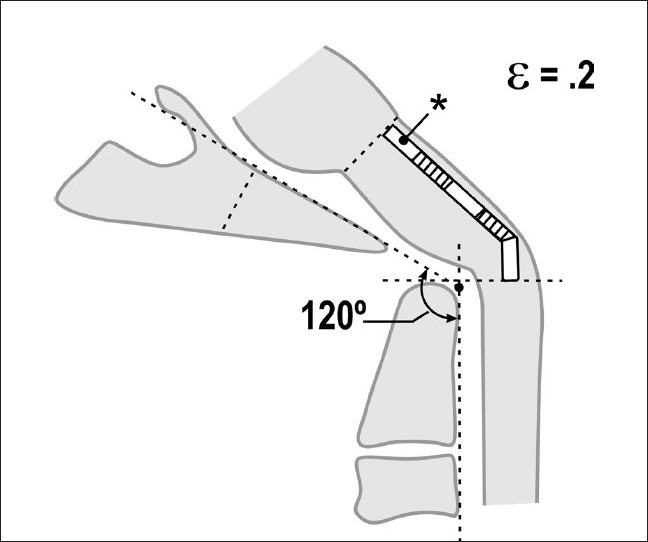
Pathological craniocervical junction with an abnormal clivo-axial angle in flexion. Upon full flexion of the craniocervical junction, mathematical analysis demonstrates that the increase in the tangent arc creates a deformative strain approaching ε = 0.2. *In vivo* and *in vitro* models demonstrate loss of function with strains of 0.2

The overall deformative stress generated at the craniocervical junction may reach levels where nerve function becomes attenuated; indeed, the axon is rendered nonconductive and develops pathological changes at a strain ε = 0.2.[72,73]

An abnormal CAA is described in the literature to manifest variously as a consequence of basilar invagination — in the setting of congenital maldevelopment of the chondrocranium, from acquired causes of bone softening in Paget’s disease, spondyloepiphyseal dysplasia, acro-osteolysis, Hurler’s syndrome, osteomalacia, Ricket’s disease, achondromalacia, renal osteodystrophy, hyperparathyroidism and in degenerative disease such as rheumatoid arthritis.[4,6,74] More subtle forms of basilar invagination have been described in which the brainstem is subject to deformation.[2,16,53] Goel suggests that some of the anatomical findings in basilar invagination, such as physical change in neck shape and length, and loss of functional range of motion, may be acquired as a protective response of the body “to minimize the stretch of the cord over the indenting odontoid process.”[75]

Building upon earlier work concerning the importance of deformative stress in the brainstem and spinal cord,[4,8,12–14,17,18,20,22,26,66,76–81] we sought to compute dynamic neuraxial stresses using FEA and images in the flexion, neutral and extension modes. FEA has been used in the spinal cord to demonstrate that stretch and strain are important determinants of pathology in cervical spondylotic myelopathy,[20] and idiopathic anterior motor neuron disease in the cervical cord.[21] To the best of our knowledge, this is the first study to undertake a mathematical modeling of the brainstem and to compute the stresses before and after surgery. The results suggest that the Von Mises stress correlates with neurological dysfunction. A decrease in the overall deformative stresses (Von Mises stresses) by the surgical techniques described, in which the CAA is normalized,[1,2,7,16] resulted in improvement in neurological function and pain.

### Pathophysiology

Clinical improvement after “relief of brainstem angulation” has been attributed to improvement in blood supply and CSF dynamics.[3] The authors make the argument that clinical improvement primarily relates to alleviation of deformative stress and substantiate this hypothesis with the growing body of neurobiological evidence.

The concept that the spinal cord elongates with flexion of the neck and that medullospinal kyphosis results in deleterious axial strain is well established.[62,66,80,81] Sawin emphasized the importance of the “fulcrum effect of the brainstem over the clivus-atlas-odontoid complex,” which places the lower brainstem and rostral spinal cord in traction.[6] Milhorat and Doherty found the same with retroflexion of the odontoid process in their Chiari series.[5,82] Nohria and Oakes noted the association of abnormal clivo-axial angle with platybasia.[67] Compression of the neuraxis causes out-of-plane loading on spinal cord axons.[83]

“Axon retraction bulbs” are the histological substrate of stretch injury in basilar invagination[12,13,84–86] and in infants with “shaken baby syndrome.”[13,76]

The importance of deformative stress in myelopathy and encephalopathy is supported in clinical reports,[13,27,66,87–89] experimental studies[77,80,85,86,90,91] and in the biomechanical[71,77,78] and mathematical literature.[20,92] The degree of injury appears to be related to the peak strain of the tissue and the loading rate. The cord is initially compliant to stretch but becomes progressively stiffer as the fibers bear tensile load.[19,93] Axonal injury relates directly to magnitude and rate of strain increase,[94] but even mild stretch can induce progressive neurofilament alteration and delayed axotomy.[95] Stretching acts upon the Na^+^ channel mechanoreceptors to increase Na^+^ influx, causing reversal of the cation exchange pumps and depolarization of voltage-gated Ca^++^ channels, with subsequent pathological influx of Ca^++^ .[95,96] Stretching of the axolemma may result in several levels of injury: decreased amplitude and increased latency, a conduction block due to myelin damage, or membrane injury with irreversible changes.[94] However, the predominant substrate for stretch-induced injury appears to be the axon. Electron micrographs show clumping, loss of microtubules and neurofilaments, loss of axon transport and accumulations of axoplasmic material identified as the “retraction ball.”[12,13,93,97–100] This pattern of axon injury in the spinal cord and brainstem is analogous to diffuse axonal injury (DAI) in the brain.[85,86] Sublethally damaged neurons also undergo up-regulation of N-methyl D-aspartate receptors, resulting in heightened vulnerability to subsequent challenges of reactive oxygen species and peroxynitrites, concomitant mitochondrial dysfunction and DNA fragmentation.[101] Early calpain activation may contribute to progressive intra-axonal structural damage after stretch injury,[73] or apoptosis of neurons and oligodendrocytes.[13,101–104] These processes are not unique to the central nervous system. Stretch injury has also been shown to induce phosphorylation of p38MAP kinase and apoptosis in vascular, heart and lung cells.[105] Clearly, the importance of biomechanical stresses upon the nervous system warrants closer attention.

### Stress modeling

Maximum stress values from the corticospinal tracts, dorsal columns, nuclei solitarius and dorsal motor nucleus were chosen to compare with clinical findings. The computed stress within each tract decreased after surgery. Moreover, stress values and measures of patient condition were always in the predicted direction — higher stress values were associated with higher pain levels and reduced SF-36 (quality of life) scores. The concordance of computed neuraxial stresses and clinical metrics support the concept that biomechanical stresses generated by stretch and “out-of-plane” loading are important determinants of neurological dysfunction.

The authors recognize that FEA modeling in the neuraxis is nascent and simplistic. The stresses are virtual computations and do not integrate measurements of stress over time and over the full length of the tract. The analysis assigns different moduli of elasticity to white and gray matter but assumes stereotypic response and uniform properties under various degrees of strain and compression. The authors have used moduli of elasticity that were described for bovine spinal cord. However, compression of the bovine cervical spinal cord produced the same histopathological changes as compression of the human cervical spinal cord,[106] and there appears to be little difference in the elastic properties between living and cadaveric spinal cord tissue.[20,107] The FEA takes into account neither the strain rate (and it does not account for alteration of compliance due to age, previous injury) nor the metabolic and circulatory factors, such as ischemia.

While other recently described systems[21,49] generate a single generic model of the human spinal cord and then simulate flexion, compression and other deformation, SCOSIA parametrically generates a unique model of both the medulla oblongata and the spinal cord for each subject, taking into account the particularities of that patient’s anatomy. SCOSIA also calculates the shear forces acting at the interface of gray and white matter tracts. However, the method for modeling each individual’s neuraxis is susceptible to user error. Until larger studies demonstrate statistically significant differences between normal and abnormal subjects, the use of FEA should be considered nonvalidated and only an approximation of stress values.

While these shortcomings clearly need to be addressed, the authors concur with others that FEA-generated stress calculations are helpful in understanding the underlying pathophysiology of a variety of spinal and brainstem conditions.[20,21,49]

### Clinical metrics and outcomes

Improvements reached statistical significance for all clinical metrics: the VAS, ASIA scale, Karnofsky Index, SF-36: physical component, SF36: mental component and the Bulbar Symptoms Index. The data presented here was collected by a research assistant. The SF-36 is a widely approved instrument for measurement of physical functioning, bodily pain, general health, vitality, social functioning and mental health. It has been shown to be valid when tested against outcome instruments.[108–110] While the ASIA scale does not measure spasticity, coordination or gait, it is useful as a metric to detect subtle changes in sensory and motor function. The Karnofsky Index was designed as a functional index for cancer patients but has also been used in other areas as a reliable means of assessing function.[111] The Bulbar Symptom Index used in this report has not yet been validated but is used by the authors to measure improvement in the panoply of symptoms generally attributed to neurological dysfunction of the brainstem.[1–7,11,16,61,67,112] A score of 100 represents significant disability [Table 1].

The statistically significant improvement of all clinical metrics viz., VAS, SF-36, Karnofsky Index, ASIA and Bulbar Symptom Index, is in agreement with the findings from the work of others — that reduction of the medullary kinking or VBSC through normalization of the craniospinal relationship, and fusion and stabilization improve neurological performance and relieve pain in subjects with traditional and “nontraditional” forms of basilar invagination.[1,2,16,113] Kim *et al*. noted excellent improvement in 10/11 patients with VBSC.[16] Salunke reported good outcomes, even amongst those with longstanding myelopathy in his series of 96 patients with atlanto-axial subluxation, suggesting that preoperative neurological changes are *not* the result of longstanding ischemic changes.[114] The observed recoverability in these injuries is consistent with the observation in experimental models — that axons subjected to strain recover rapidly.[94]

### Comorbidities

The authors emphasize the breadth of associated comorbidities in this small cohort — respiratory disorders and gastroesophageal reflux disease, personality disorders, leg tremors, tongue protrusion (“trombone tongue”) and scoliosis [Table 2]. These comorbidities resolved or substantially improved following the craniocervical surgery. The literature supports this observation. Respiratory and gastrointestinal disorders are reported with a prevalence of 10% to 17% of patients with craniocervical disorders.[27,64,76,114–117] Gastroesophageal disorders are known concomitants to compressive deformity of the dorsal motor nuclei.[118–121] Resolution of a condition of “trombone tongue” has been reported with treatment of basilar invagination.[122] Scoliosis in Chiari subjects often resolves after surgical treatment of the Chiari malformation.[123,124] While psychological evaluation of these children was beyond the scope of this manuscript, the improvement in behavior warrants evaluation of a larger cohort of subjects diagnosed with behavioral disorders to determine the extent of overlap of cervicomedullary syndromes with abnormal psychological profile.

### Surgical technique

The authors performed open reduction with manual distraction and extension of the cranium, as described by Kim *et al*.[16] The need for transoral odontoidectomy[2,74,112,125,126] is obviated in many cases by open reduction with manual distraction and extension at the craniocervical junction.[16] A posterior translation of the cranium with respect to the spine and an alignment of the Basion over the tip of the dens was achieved by manual manipulation during surgery. Patients tolerate the surgery well.[2,7,9,11,16,74,113]

### Complications

Postoperatively, a C2 pedicle screw was observed to be adjacent to the vertebral artery in 1 patient (patient # 2), in whom the subsequent MRA was normal. The headaches in patient # 4 were thought to be due to occipital neuralgia. Hence the authors recommend placement of C1 screws in a manner that avoids encumbrance of the exiting C2 roots.

Kim *et al*. reported a 36% complication rate, but with the exception of 1 patient in whom hyperostosis necessitated a posterior decompression, these were minor complications.[16] Kumar reported 2 deaths due to spinal cord injury, sustained when the patient was turned to the prone position.[74]

A concern in children is the limitation of neck rotation after craniospinal fusion. Fifty percent of neck rotation occurs between C1 and C2, and approximately 21° of flexion is observed between the occiput and cervical spine.[70] However, only 1 patient of this group complained of decreased neck rotation; the remainder reported nearly normal movement at 1 year, presumably as a result of increased rotation of lower cervical levels, compensatory torso rotation[114] and Toyama remodeling of vertebrae.[127] We are unaware of delayed complications of occipitocervical fusion in children, when fusion is limited to the upper two cervical vertebrae.

The authors emphasize the risk associated with injury to the vertebral artery.[128–130] The published data for craniospinal fusion stabilization shows that the morbidity of this operation compares favorably with other common spinal surgeries, such as lumbar discectomy.[131]

## CONCLUSION

Conventional radiographic assessment of basilar invagination does not reveal the more subtle forms of ventral brainstem compression and deformation. Open reduction of craniospinal deformity (normalization of clivo-axial angle), stabilization and surgical fusion are effective in improving pain and neurological function in subjects with cervicomedullary disorders resulting from deformative stress. The growing body of neurobiological literature supports the concept that deformative stress is important in both direct and epigenetic mechanisms of neurological dysfunction.

We have used FEA to compute neuraxial deformative stress in the context of cervicomedullary disorders due to Chiari malformation and basilar invagination. Surgical correction of the deformity resulted in improvement of computed Von Mises stress in selected anatomical structures, which was concordant with relief of pain and neurological deficits. FEA may offer new insight into the effect of pressure and strain on the neuraxis at the cervicomedullary junction. Further investigations are warranted to validate the concept that deformative stress is an important determinant of neurological function.
